# Editorial: Autophagy in Autoimmunity

**DOI:** 10.3389/fimmu.2019.00301

**Published:** 2019-02-25

**Authors:** Xu-jie Zhou, Panayotis Verginis, Jennifer Martinez, Marko Radic

**Affiliations:** ^1^Renal Division, Peking University First Hospital, Beijing, China; ^2^Peking University Institute of Nephrology, Beijing, China; ^3^Key Laboratory of Renal Disease, Ministry of Health of China, Beijing, China; ^4^Key Laboratory of Chronic Kidney Disease Prevention and Treatment (Peking University), Ministry of Education, Beijing, China; ^5^Biomedical Research Foundation of the Academy of Athens, Athens, Greece; ^6^National Institute of Environmental Health Sciences, Durham, NC, United States; ^7^Department of Microbiology, Immunology and Biochemistry, College of Medicine, University of Tennessee Health Science Center, Memphis, TN, United States

**Keywords:** autophagy, autoimmunity, immunity, translational, human disease

In this Research Topic, we hosted several in-depth reviews, minireviews, and original research articles on the role of autophagy in autoimmunity.

Autophagy is an important process involved in the growth, development, physiology, and pathology of cells. It is a “double-edged sword”—exerting physiologically necessary and therapeutically useful but also potentially detrimental effects on cells and tissues. These contrasting outcomes may be accounted for by differences in the type of autophagy, the tissue or time of induction, activation mode, and stress severity. This ancient set of pathways, conserved from yeast to humans, is now emerging as a central player in nearly all aspects of immunity. Although our knowledge of the molecular mechanisms of autophagy in relation to immune-related disease has been greatly improved in recent years, additional information is needed to fully understand the role of autophagy in clinical settings and to target this set of pathways. Of importance, autophagy has been demonstrated to participate in various processes of the immune response from antigen processing to antigen presentation and therefore should be consider as an important node in the activation and expansion of autoreactive clones. Therefore, we must continue to uncover the pathogenesis and regulation of autophagy in various autoimmune diseases in order to identify new diagnostic and therapeutic approaches.

This Research Topic highlights the essential roles of autophagy in human and animal model tissues that are affected in various autoimmune diseases, including skin, lung, liver, bone, cardiovascular, and intestine systems. Arbogast and Gros discuss the mechanisms linking autophagy to lymphocyte subtype survival and the signaling pathways involved to elaborate the function of autophagy in autoimmune pathology. Reviews by Ye et al. and Hargarten and Williamson illustrate how the function of autophagy-related gene ATG5 and the epigenetic modulation of histones affect RNA transcription and half-life, miRNA expression, and directly impact immunological function that affect human autoimmune diseases. These data from targeted autophagic gene models are important and necessary to elucidate and clarify the precise functional role of autophagy in the etiology of autoimmune diseases and potentially guide future therapeutic applications. Apart from classical autophagy, Gkikas et al. showcase how mitochondrial selective autophagy (mitophagy) connects to cellular health and how its deregulation leads to impaired mitochondrial metabolism and inflammatory disorders. Sil et al. review the potential connection between the autophagic machinery and the homeostasis of skin cells (both immune and non-immune cells), in order to trace the consequences of its disruption by infections, or mechanical damage that may ultimately lead to autoimmunity. Vomero et al. elaborate how autophagy activation is involved in the pathogenesis of rheumatoid arthritis (RA) by highlighting how autophagy contributes to the survival of synoviocytes and lymphocytes, and how autophagy is implicated in protein citrullination and osteoclastogenesis. Yin et al. address the different roles of autophagy in different autoimmune diseases and recommend future approaches to personalized therapy that consider judicious regulation of autophagy.

In mice, Amersfoort et al. explore the effects of a T cell-specific knock-out of Atg7 (Lck-Cre Atg7^f/f^) on CD4+, CD8+, and NKT cells and observe a decrease in the induction of hepatic steatosis and atherosclerosis. Bendorius et al. report on experiments with the phosphopeptide P140 (Lupuzor), an inhibitor of autophagy, which shows promise for treating patients with lupus and targets chaperone-mediated autophagy (CMA) and macroautophagy rather than mitophagy. A separate effect, the inhibition of neutrophil extracellular trap (NET) release by P140 was also described. Feng et al. demonstrate that autophagy is deregulated in concanavalin A-induced autoimmune hepatitis and that the plant lignin arctigenin inhibits the IFN-γ/IL-6/Stat1 and the IL-6/Bnip3 pathways that are activated in this model of hepatic injury. Thus, arctigenin may have great therapeutic potential in immune-mediated hepatitis. Lin et al. observe that excessive mechanical ventilation with high tidal volumes triggers mitochondrial damage in lungs of rats, which activates mitophagy, results in mitochondrial membrane fracture and mtDNA release, and, ultimately promotes inflammation and injury. These interesting original research studies and expert reviews were indicate that autophagy participates in various immune related injuries. Undoubtedly, the collection of articles also supports the view that autophagy is an attractive target for developing new treatments for immune-mediated disorders.

We are happy to see the issue is welcomed in the field and the aspect of autophagy garnered significant interest from both biomarker, translational, and therapeutic standpoint.

However, at the current stage, the precise contribution of autophagy to the induction and progression of autoimmunity is still not well-understood. There have been studies that provide evidence to support a cytoprotective role of autophagy, and others that support deleterious effects of autophagy. It is plausible that the outcome is context dependent. The difference in the types of injuries and severity of injuries may produce different outcome of autophagy, in that, a certain degree of autophagic activity can maintain tissue homeostasis, whereas excessive autophagic activity results in cell death. Future investigations, for instance by using targeted autophagic gene knockout mice, and novel and systems biology assays, are still necessary.

## Author Contributions

All authors listed have made a substantial, direct and intellectual contribution to the work, and approved it for publication.

### Conflict of Interest Statement

The authors declare that the research was conducted in the absence of any commercial or financial relationships that could be construed as a potential conflict of interest.

